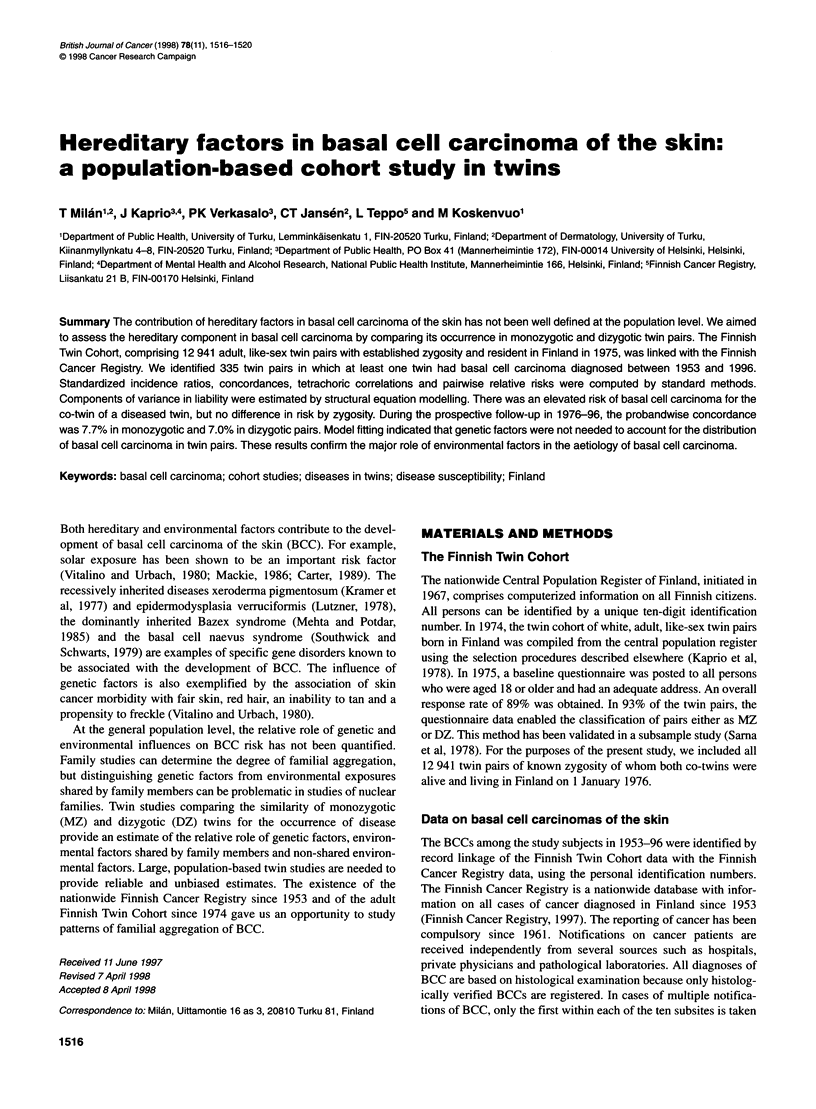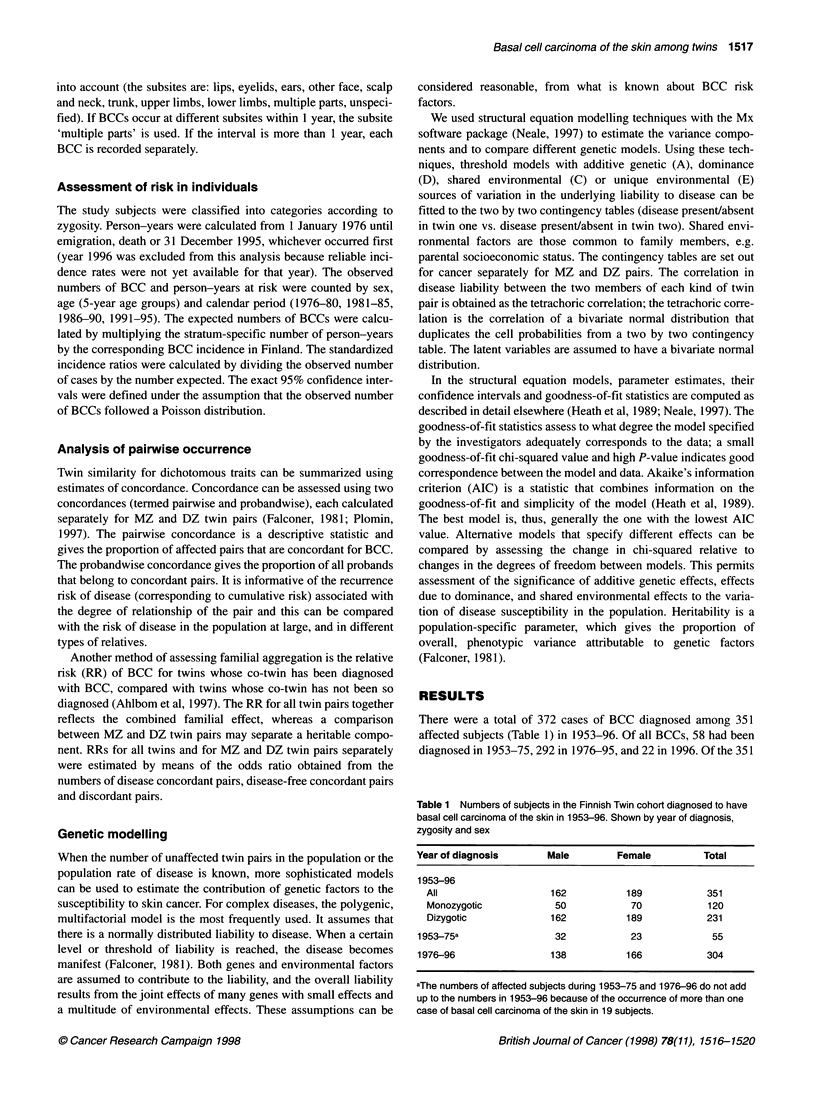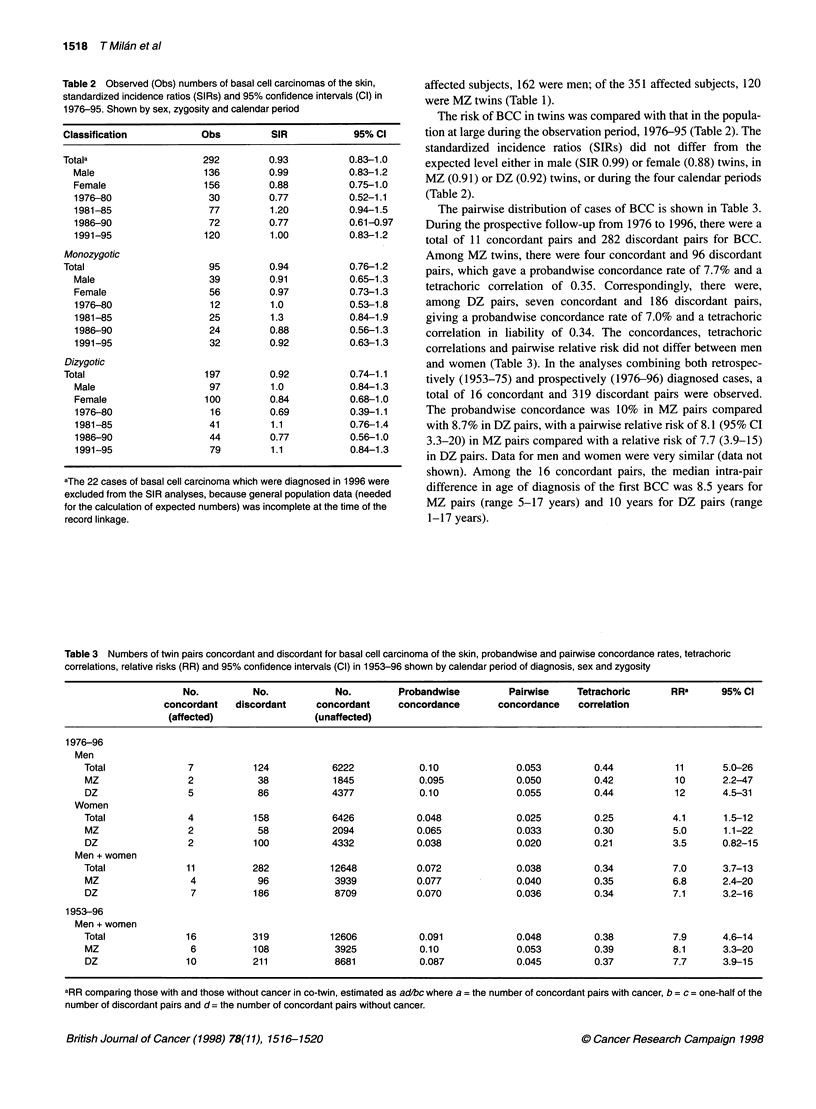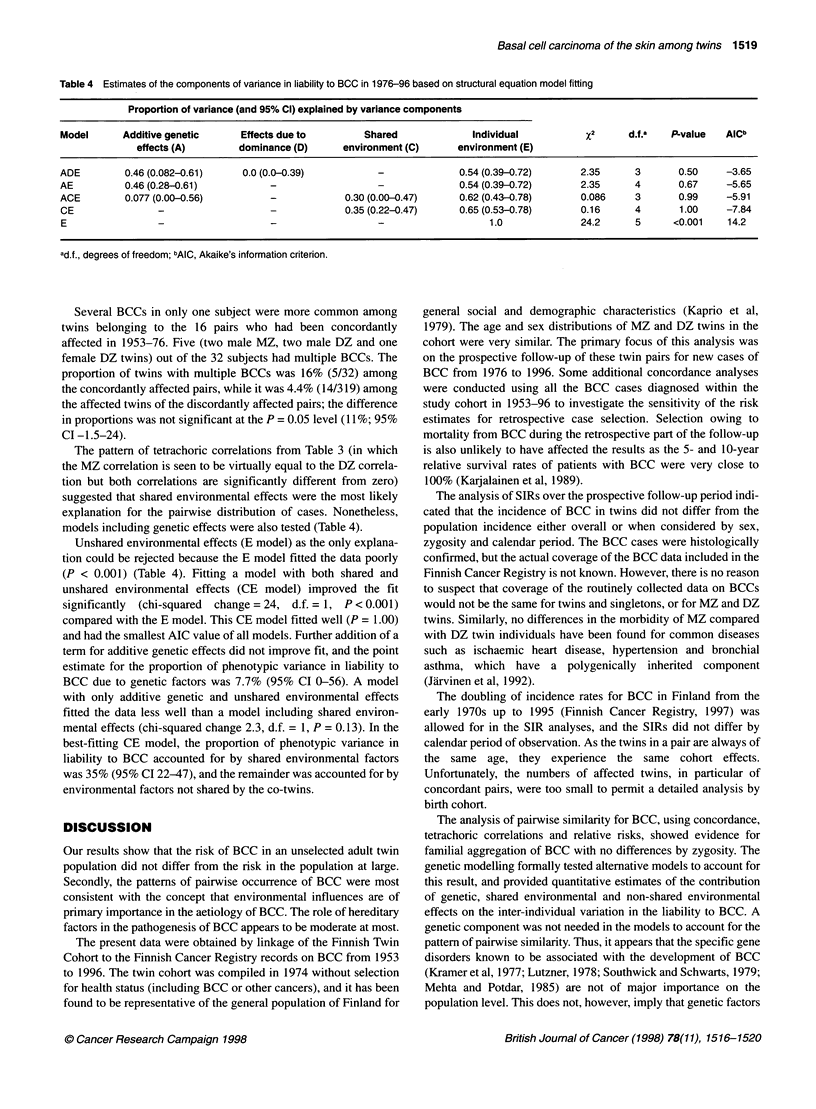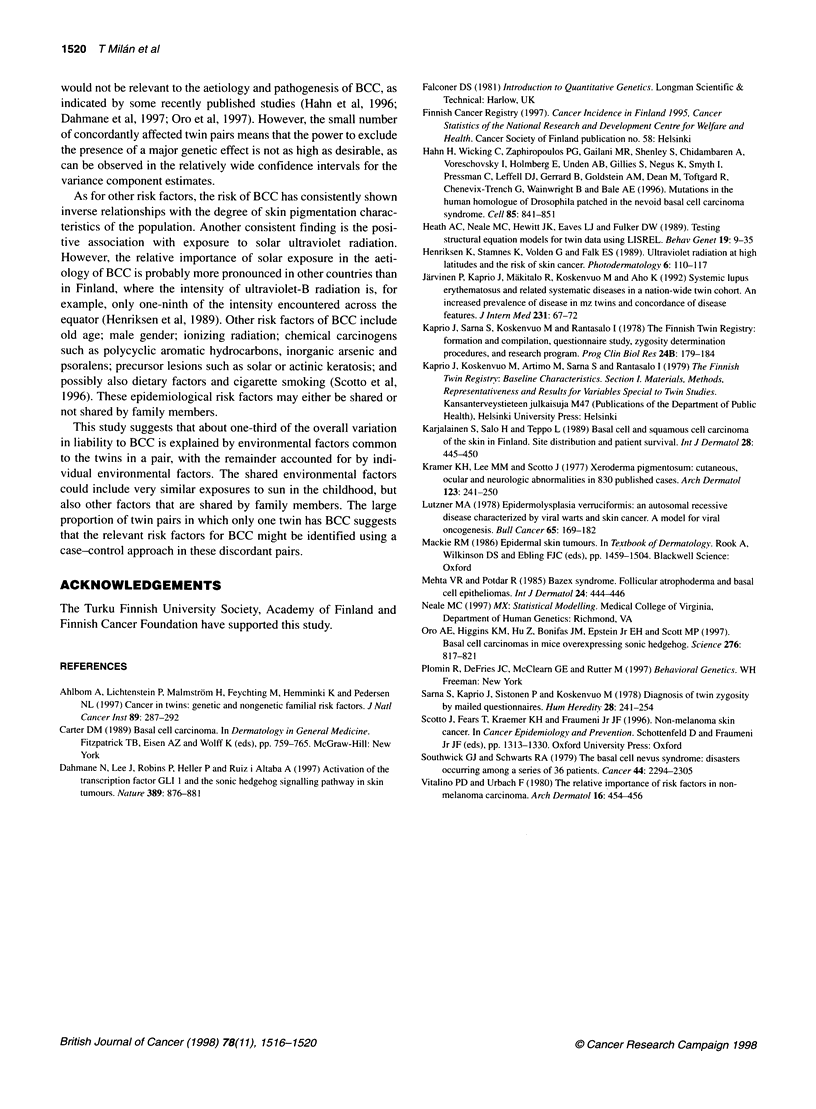# Hereditary factors in basal cell carcinoma of the skin: a population-based cohort study in twins.

**DOI:** 10.1038/bjc.1998.716

**Published:** 1998-12

**Authors:** T. Milán, J. Kaprio, P. K. Verkasalo, C. T. Jansén, L. Teppo, M. Koskenvuo

**Affiliations:** Department of Public Health, University of Turku, Finland.

## Abstract

The contribution of hereditary factors in basal cell carcinoma of the skin has not been well defined at the population level. We aimed to assess the hereditary component in basal cell carcinoma by comparing its occurrence in monozygotic and dizygotic twin pairs. The Finnish Twin Cohort, comprising 12,941 adult, like-sex twin pairs with established zygosity and resident in Finland in 1975, was linked with the Finnish Cancer Registry. We identified 335 twin pairs in which at least one twin had basal cell carcinoma diagnosed between 1953 and 1996. Standardized incidence ratios, concordances, tetrachoric correlations and pairwise relative risks were computed by standard methods. Components of variance in liability were estimated by structural equation modelling. There was an elevated risk of basal cell carcinoma for the co-twin of a diseased twin, but no difference in risk by zygosity. During the prospective follow-up in 1976-96, the probandwise concordance was 7.7% in monozygotic and 7.0% in dizygotic pairs. Model fitting indicated that genetic factors were not needed to account for the distribution of basal cell carcinoma in twin pairs. These results confirm the major role of environmental factors in the aetiology of basal cell carcinoma.


British Journal of Cancer (1998) 78(11), 1516–1520
© 1998 Cancer Research Campaign

### Hereditary factors in basal cell carcinoma of the skin: a population-based cohort study in twins

**T Milán[1,2], J Kaprio[3,4], PK Verkasalo[3], CT Jansén[2], L Teppo[5] and M Koskenvuo[1]**

[1]Department of Public Health, University of Turku, Lemminkäisenkatu 1, FIN-20520 Turku, Finland; [2]Department of Dermatology, University of Turku, Kiinanmyllynkatu 4–8, FIN-20520 Turku, Finland; [3]Department of Public Health, PO Box 41 (Mannerheimintie 172), FIN-00014 University of Helsinki, Helsinki, Finland; [4]Department of Mental Health and Alcohol Research, National Public Health Institute, Mannerheimintie 166, Helsinki, Finland; [5]Finnish Cancer Registry, Liisankatu 21 B, FIN-00170 Helsinki, Finland

**Summary** The contribution of hereditary factors in basal cell carcinoma of the skin has not been well defined at the population level. We aimed to assess the hereditary component in basal cell carcinoma by comparing its occurrence in monozygotic and dizygotic twin pairs. The Finnish Twin Cohort, comprising 12 941 adult, like-sex twin pairs with established zygosity and resident in Finland in 1975, was linked with the Finnish Cancer Registry. We identified 335 twin pairs in which at least one twin had basal cell carcinoma diagnosed between 1953 and 1996. Standardized incidence ratios, concordances, tetrachoric correlations and pairwise relative risks were computed by standard methods. Components of variance in liability were estimated by structural equation modelling. There was an elevated risk of basal cell carcinoma for the co-twin of a diseased twin, but no difference in risk by zygosity. During the prospective follow-up in 1976–96, the probandwise concordance was 7.7% in monozygotic and 7.0% in dizygotic pairs. Model fitting indicated that genetic factors were not needed to account for the distribution of basal cell carcinoma in twin pairs. These results confirm the major role of environmental factors in the aetiology of basal cell carcinoma.

**Keywords:** basal cell carcinoma; cohort studies; diseases in twins; disease susceptibility; Finland

Both hereditary and environmental factors contribute to the development of basal cell carcinoma of the skin (BCC). For example, solar exposure has been shown to be an important risk factor (Vitalino and Urbach, 1980; Mackie, 1986; Carter, 1989). The recessively inherited diseases xeroderma pigmentosum (Kramer et al, 1977) and epidermodysplasia verruciformis (Lutzner, 1978), the dominantly inherited Bazex syndrome (Mehta and Potdar, 1985) and the basal cell naevus syndrome (Southwick and Schwarts, 1979) are examples of specific gene disorders known to be associated with the development of BCC. The influence of genetic factors is also exemplified by the association of skin cancer morbidity with fair skin, red hair, an inability to tan and a propensity to freckle (Vitalino and Urbach, 1980).

At the general population level, the relative role of genetic and environmental influences on BCC risk has not been quantified. Family studies can determine the degree of familial aggregation, but distinguishing genetic factors from environmental exposures shared by family members can be problematic in studies of nuclear families. Twin studies comparing the similarity of monozygotic (MZ) and dizygotic (DZ) twins for the occurrence of disease provide an estimate of the relative role of genetic factors, environmental factors shared by family members and non-shared environmental factors. Large, population-based twin studies are needed to provide reliable and unbiased estimates. The existence of the nationwide Finnish Cancer Registry since 1953 and of the adult Finnish Twin Cohort since 1974 gave us an opportunity to study patterns of familial aggregation of BCC.

*Received 11 June 1997*
*Revised 7 April 1998*
*Accepted 8 April 1998*

*Correspondence to:* Milán, Uittamontie 16 as 3, 20810 Turku 81, Finland

### MATERIALS AND METHODS

### The Finnish Twin Cohort

The nationwide Central Population Register of Finland, initiated in 1967, comprises computerized information on all Finnish citizens. All persons can be identified by a unique ten-digit identification number. In 1974, the twin cohort of white, adult, like-sex twin pairs born in Finland was compiled from the central population register using the selection procedures described elsewhere (Kaprio et al, 1978). In 1975, a baseline questionnaire was posted to all persons who were aged 18 or older and had an adequate address. An overall response rate of 89% was obtained. In 93% of the twin pairs, the questionnaire data enabled the classification of pairs either as MZ or DZ. This method has been validated in a subsample study (Sarna et al, 1978). For the purposes of the present study, we included all 12 941 twin pairs of known zygosity of whom both co-twins were alive and living in Finland on 1 January 1976.

### Data on basal cell carcinomas of the skin

The BCCs among the study subjects in 1953–96 were identified by record linkage of the Finnish Twin Cohort data with the Finnish Cancer Registry data, using the personal identification numbers. The Finnish Cancer Registry is a nationwide database with information on all cases of cancer diagnosed in Finland since 1953 (Finnish Cancer Registry, 1997). The reporting of cancer has been compulsory since 1961. Notifications on cancer patients are received independently from several sources such as hospitals, private physicians and pathological laboratories. All diagnoses of BCC are based on histological examination because only histologically verified BCCs are registered. In cases of multiple notifications of BCC, only the first within each of the ten subsites is taken into account (the subsites are: lips, eyelids, ears, other face, scalp and neck, trunk, upper limbs, lower limbs, multiple parts, unspecified). If BCCs occur at different subsites within 1 year, the subsite 'multiple parts' is used. If the interval is more than 1 year, each BCC is recorded separately.

### Assessment of risk in individuals

The study subjects were classified into categories according to zygosity. Person–years were calculated from 1 January 1976 until emigration, death or 31 December 1995, whichever occurred first (year 1996 was excluded from this analysis because reliable incidence rates were not yet available for that year). The observed numbers of BCC and person–years at risk were counted by sex, age (5-year age groups) and calendar period (1976–80, 1981–85, 1986–90, 1991–95). The expected numbers of BCCs were calculated by multiplying the stratum-specific number of person–years by the corresponding BCC incidence in Finland. The standardized incidence ratios were calculated by dividing the observed number of cases by the number expected. The exact 95% confidence intervals were defined under the assumption that the observed number of BCCs followed a Poisson distribution.

### Analysis of pairwise occurrence

Twin similarity for dichotomous traits can be summarized using estimates of concordance. Concordance can be assessed using two concordances (termed pairwise and probandwise), each calculated separately for MZ and DZ twin pairs (Falconer, 1981; Plomin, 1997). The pairwise concordance is a descriptive statistic and gives the proportion of affected pairs that are concordant for BCC. The probandwise concordance gives the proportion of all probands that belong to concordant pairs. It is informative of the recurrence risk of disease (corresponding to cumulative risk) associated with the degree of relationship of the pair and this can be compared with the risk of disease in the population at large, and in different types of relatives.

Another method of assessing familial aggregation is the relative risk (RR) of BCC for twins whose co-twin has been diagnosed with BCC, compared with twins whose co-twin has not been so diagnosed (Ahlbom et al, 1997). The RR for all twin pairs together reflects the combined familial effect, whereas a comparison between MZ and DZ twin pairs may separate a heritable component. RRs for all twins and for MZ and DZ twin pairs separately were estimated by means of the odds ratio obtained from the numbers of disease concordant pairs, disease-free concordant pairs and discordant pairs.

### Genetic modelling

When the number of unaffected twin pairs in the population or the population rate of disease is known, more sophisticated models can be used to estimate the contribution of genetic factors to the susceptibility to skin cancer. For complex diseases, the polygenic, multifactorial model is the most frequently used. It assumes that there is a normally distributed liability to disease. When a certain level or threshold of liability is reached, the disease becomes manifest (Falconer, 1981). Both genes and environmental factors are assumed to contribute to the liability, and the overall liability results from the joint effects of many genes with small effects and a multitude of environmental effects. These assumptions can be considered reasonable, from what is known about BCC risk factors.

We used structural equation modelling techniques with the Mx software package (Neale, 1997) to estimate the variance components and to compare different genetic models. Using these techniques, threshold models with additive genetic (A), dominance (D), shared environmental (C) or unique environmental (E) sources of variation in the underlying liability to disease can be fitted to the two by two contingency tables (disease present/absent in twin one vs. disease present/absent in twin two). Shared environmental factors are those common to family members, e.g. parental socioeconomic status. The contingency tables are set out for cancer separately for MZ and DZ pairs. The correlation in disease liability between the two members of each kind of twin pair is obtained as the tetrachoric correlation; the tetrachoric correlation is the correlation of a bivariate normal distribution that duplicates the cell probabilities from a two by two contingency table. The latent variables are assumed to have a bivariate normal distribution.

In the structural equation models, parameter estimates, their confidence intervals and goodness-of-fit statistics are computed as described in detail elsewhere (Heath et al, 1989; Neale, 1997). The goodness-of-fit statistics assess to what degree the model specified by the investigators adequately corresponds to the data; a small goodness-of-fit chi-squared value and high $P$-value indicates good correspondence between the model and data. Akaike's information criterion (AIC) is a statistic that combines information on the goodness-of-fit and simplicity of the model (Heath et al, 1989). The best model is, thus, generally the one with the lowest AIC value. Alternative models that specify different effects can be compared by assessing the change in chi-squared relative to changes in the degrees of freedom between models. This permits assessment of the significance of additive genetic effects, effects due to dominance, and shared environmental effects to the variation of disease susceptibility in the population. Heritability is a population-specific parameter, which gives the proportion of overall, phenotypic variance attributable to genetic factors (Falconer, 1981).

### RESULTS

There were a total of 372 cases of BCC diagnosed among 351 affected subjects (Table 1) in 1953–96. Of all BCCs, 58 had been diagnosed in 1953–75, 292 in 1976–95, and 22 in 1996. Of the 351

**Table 1** Numbers of subjects in the Finnish Twin cohort diagnosed to have basal cell carcinoma of the skin in 1953–96. Shown by year of diagnosis, zygosity and sex

| Year of diagnosis | Male | Female | Total |
|---|---|---|---|
| 1953–96 | | | |
| All | 162 | 189 | 351 |
| Monozygotic | 50 | 70 | 120 |
| Dizygotic | 162 | 189 | 231 |
| 1953–75[a] | 32 | 23 | 55 |
| 1976–96 | 138 | 166 | 304 |

[a]The numbers of affected subjects during 1953–75 and 1976–96 do not add up to the numbers in 1953–96 because of the occurrence of more than one case of basal cell carcinoma of the skin in 19 subjects.

© Cancer Research Campaign 1998

**Table 2** Observed (Obs) numbers of basal cell carcinomas of the skin, standardized incidence ratios (SIRs) and 95% confidence intervals (CI) in 1976–95. Shown by sex, zygosity and calendar period

| Classification | Obs | SIR | 95% CI |
|---|---|---|---|
| Total[a] | 292 | 0.93 | 0.83–1.0 |
| Male | 136 | 0.99 | 0.83–1.2 |
| Female | 156 | 0.88 | 0.75–1.0 |
| 1976–80 | 30 | 0.77 | 0.52–1.1 |
| 1981–85 | 77 | 1.20 | 0.94–1.5 |
| 1986–90 | 72 | 0.77 | 0.61–0.97 |
| 1991–95 | 120 | 1.00 | 0.83–1.2 |
| *Monozygotic* | | | |
| Total | 95 | 0.94 | 0.76–1.2 |
| Male | 39 | 0.91 | 0.65–1.3 |
| Female | 56 | 0.97 | 0.73–1.3 |
| 1976–80 | 12 | 1.0 | 0.53–1.8 |
| 1981–85 | 25 | 1.3 | 0.84–1.9 |
| 1986–90 | 24 | 0.88 | 0.56–1.3 |
| 1991–95 | 32 | 0.92 | 0.63–1.3 |
| *Dizygotic* | | | |
| Total | 197 | 0.92 | 0.74–1.1 |
| Male | 97 | 1.0 | 0.84–1.3 |
| Female | 100 | 0.84 | 0.68–1.0 |
| 1976–80 | 16 | 0.69 | 0.39–1.1 |
| 1981–85 | 41 | 1.1 | 0.76–1.4 |
| 1986–90 | 44 | 0.77 | 0.56–1.0 |
| 1991–95 | 79 | 1.1 | 0.84–1.3 |

[a]The 22 cases of basal cell carcinoma which were diagnosed in 1996 were excluded from the SIR analyses, because general population data (needed for the calculation of expected numbers) was incomplete at the time of the record linkage.

affected subjects, 162 were men; of the 351 affected subjects, 120 were MZ twins (Table 1).

The risk of BCC in twins was compared with that in the population at large during the observation period, 1976–95 (Table 2). The standardized incidence ratios (SIRs) did not differ from the expected level either in male (SIR 0.99) or female (0.88) twins, in MZ (0.91) or DZ (0.92) twins, or during the four calendar periods (Table 2).

The pairwise distribution of cases of BCC is shown in Table 3. During the prospective follow-up from 1976 to 1996, there were a total of 11 concordant pairs and 282 discordant pairs for BCC. Among MZ twins, there were four concordant and 96 discordant pairs, which gave a probandwise concordance rate of 7.7% and a tetrachoric correlation of 0.35. Correspondingly, there were, among DZ pairs, seven concordant and 186 discordant pairs, giving a probandwise concordance rate of 7.0% and a tetrachoric correlation in liability of 0.34. The concordances, tetrachoric correlations and pairwise relative risk did not differ between men and women (Table 3). In the analyses combining both retrospectively (1953–75) and prospectively (1976–96) diagnosed cases, a total of 16 concordant and 319 discordant pairs were observed. The probandwise concordance was 10% in MZ pairs compared with 8.7% in DZ pairs, with a pairwise relative risk of 8.1 (95% CI 3.3–20) in MZ pairs compared with a relative risk of 7.7 (3.9–15) in DZ pairs. Data for men and women were very similar (data not shown). Among the 16 concordant pairs, the median intra-pair difference in age of diagnosis of the first BCC was 8.5 years for MZ pairs (range 5–17 years) and 10 years for DZ pairs (range 1–17 years).

**Table 3** Numbers of twin pairs concordant and discordant for basal cell carcinoma of the skin, probandwise and pairwise concordance rates, tetrachoric correlations, relative risks (RR) and 95% confidence intervals (CI) in 1953–96 shown by calendar period of diagnosis, sex and zygosity

| | No. concordant (affected) | No. discordant | No. concordant (unaffected) | Probandwise concordance | Pairwise concordance | Tetrachoric correlation | RR[a] | 95% CI |
|---|---|---|---|---|---|---|---|---|
| 1976–96 | | | | | | | | |
| Men | | | | | | | | |
| Total | 7 | 124 | 6222 | 0.10 | 0.053 | 0.44 | 11 | 5.0–26 |
| MZ | 2 | 38 | 1845 | 0.095 | 0.050 | 0.42 | 10 | 2.2–47 |
| DZ | 5 | 86 | 4377 | 0.10 | 0.055 | 0.44 | 12 | 4.5–31 |
| Women | | | | | | | | |
| Total | 4 | 158 | 6426 | 0.048 | 0.025 | 0.25 | 4.1 | 1.5–12 |
| MZ | 2 | 58 | 2094 | 0.065 | 0.033 | 0.30 | 5.0 | 1.1–22 |
| DZ | 2 | 100 | 4332 | 0.038 | 0.020 | 0.21 | 3.5 | 0.82–15 |
| Men + women | | | | | | | | |
| Total | 11 | 282 | 12648 | 0.072 | 0.038 | 0.34 | 7.0 | 3.7–13 |
| MZ | 4 | 96 | 3939 | 0.077 | 0.040 | 0.35 | 6.8 | 2.4–20 |
| DZ | 7 | 186 | 8709 | 0.070 | 0.036 | 0.34 | 7.1 | 3.2–16 |
| 1953–96 | | | | | | | | |
| Men + women | | | | | | | | |
| Total | 16 | 319 | 12606 | 0.091 | 0.048 | 0.38 | 7.9 | 4.6–14 |
| MZ | 6 | 108 | 3925 | 0.10 | 0.053 | 0.39 | 8.1 | 3.3–20 |
| DZ | 10 | 211 | 8681 | 0.087 | 0.045 | 0.37 | 7.7 | 3.9–15 |

[a]RR comparing those with and those without cancer in co-twin, estimated as $ad/bc$ where $a$ = the number of concordant pairs with cancer, $b = c$ = one-half of the number of discordant pairs and $d$ = the number of concordant pairs without cancer.

*© Cancer Research Campaign 1998*

**Table 4** Estimates of the components of variance in liability to BCC in 1976–96 based on structural equation model fitting

| Model | Proportion of variance (and 95% CI) explained by variance components | | | | $\chi^2$ | d.f.[a] | P-value | AIC[b] |
|---|---|---|---|---|---|---|---|---|
| | Additive genetic effects (A) | Effects due to dominance (D) | Shared environment (C) | Individual environment (E) | | | | |
| ADE | 0.46 (0.082–0.61) | 0.0 (0.0–0.39) | – | 0.54 (0.39–0.72) | 2.35 | 3 | 0.50 | –3.65 |
| AE | 0.46 (0.28–0.61) | – | – | 0.54 (0.39–0.72) | 2.35 | 4 | 0.67 | –5.65 |
| ACE | 0.077 (0.00–0.56) | – | 0.30 (0.00–0.47) | 0.62 (0.43–0.78) | 0.086 | 3 | 0.99 | –5.91 |
| CE | – | – | 0.35 (0.22–0.47) | 0.65 (0.53–0.78) | 0.16 | 4 | 1.00 | –7.84 |
| E | – | – | – | 1.0 | 24.2 | 5 | <0.001 | 14.2 |

[a]d.f., degrees of freedom; [b]AIC, Akaike's information criterion.

Several BCCs in only one subject were more common among twins belonging to the 16 pairs who had been concordantly affected in 1953–76. Five (two male MZ, two male DZ and one female DZ twins) out of the 32 subjects had multiple BCCs. The proportion of twins with multiple BCCs was 16% (5/32) among the concordantly affected pairs, while it was 4.4% (14/319) among the affected twins of the discordantly affected pairs; the difference in proportions was not significant at the $P = 0.05$ level (11%; 95% CI -1.5–24).

The pattern of tetrachoric correlations from Table 3 (in which the MZ correlation is seen to be virtually equal to the DZ correlation but both correlations are significantly different from zero) suggested that shared environmental effects were the most likely explanation for the pairwise distribution of cases. Nonetheless, models including genetic effects were also tested (Table 4).

Unshared environmental effects (E model) as the only explanation could be rejected because the E model fitted the data poorly ($P < 0.001$) (Table 4). Fitting a model with both shared and unshared environmental effects (CE model) improved the fit significantly (chi-squared change = 24, d.f. = 1, $P < 0.001$) compared with the E model. This CE model fitted well ($P = 1.00$) and had the smallest AIC value of all models. Further addition of a term for additive genetic effects did not improve fit, and the point estimate for the proportion of phenotypic variance in liability to BCC due to genetic factors was 7.7% (95% CI 0–56). A model with only additive genetic and unshared environmental effects fitted the data less well than a model including shared environmental effects (chi-squared change 2.3, d.f. = 1, $P = 0.13$). In the best-fitting CE model, the proportion of phenotypic variance in liability to BCC accounted for by shared environmental factors was 35% (95% CI 22–47), and the remainder was accounted for by environmental factors not shared by the co-twins.

### DISCUSSION

Our results show that the risk of BCC in an unselected adult twin population did not differ from the risk in the population at large. Secondly, the patterns of pairwise occurrence of BCC were most consistent with the concept that environmental influences are of primary importance in the aetiology of BCC. The role of hereditary factors in the pathogenesis of BCC appears to be moderate at most.

The present data were obtained by linkage of the Finnish Twin Cohort to the Finnish Cancer Registry records on BCC from 1953 to 1996. The twin cohort was compiled in 1974 without selection for health status (including BCC or other cancers), and it has been found to be representative of the general population of Finland for general social and demographic characteristics (Kaprio et al, 1979). The age and sex distributions of MZ and DZ twins in the cohort were very similar. The primary focus of this analysis was on the prospective follow-up of these twin pairs for new cases of BCC from 1976 to 1996. Some additional concordance analyses were conducted using all the BCC cases diagnosed within the study cohort in 1953–96 to investigate the sensitivity of the risk estimates for retrospective case selection. Selection owing to mortality from BCC during the retrospective part of the follow-up is also unlikely to have affected the results as the 5- and 10-year relative survival rates of patients with BCC were very close to 100% (Karjalainen et al, 1989).

The analysis of SIRs over the prospective follow-up period indicated that the incidence of BCC in twins did not differ from the population incidence either overall or when considered by sex, zygosity and calendar period. The BCC cases were histologically confirmed, but the actual coverage of the BCC data included in the Finnish Cancer Registry is not known. However, there is no reason to suspect that coverage of the routinely collected data on BCCs would not be the same for twins and singletons, or for MZ and DZ twins. Similarly, no differences in the morbidity of MZ compared with DZ twin individuals have been found for common diseases such as ischaemic heart disease, hypertension and bronchial asthma, which have a polygenically inherited component (Järvinen et al, 1992).

The doubling of incidence rates for BCC in Finland from the early 1970s up to 1995 (Finnish Cancer Registry, 1997) was allowed for in the SIR analyses, and the SIRs did not differ by calendar period of observation. As the twins in a pair are always of the same age, they experience the same cohort effects. Unfortunately, the numbers of affected twins, in particular of concordant pairs, were too small to permit a detailed analysis by birth cohort.

The analysis of pairwise similarity for BCC, using concordance, tetrachoric correlations and relative risks, showed evidence for familial aggregation of BCC with no differences by zygosity. The genetic modelling formally tested alternative models to account for this result, and provided quantitative estimates of the contribution of genetic, shared environmental and non-shared environmental effects on the inter-individual variation in the liability to BCC. A genetic component was not needed in the models to account for the pattern of pairwise similarity. Thus, it appears that the specific gene disorders known to be associated with the development of BCC (Kramer et al, 1977; Lutzner, 1978; Southwick and Schwarts, 1979; Mehta and Potdar, 1985) are not of major importance on the population level. This does not, however, imply that genetic factors

© Cancer Research Campaign 1998

would not be relevant to the aetiology and pathogenesis of BCC, as indicated by some recently published studies (Hahn et al, 1996; Dahmane et al, 1997; Oro et al, 1997). However, the small number of concordantly affected twin pairs means that the power to exclude the presence of a major genetic effect is not as high as desirable, as can be observed in the relatively wide confidence intervals for the variance component estimates.

As for other risk factors, the risk of BCC has consistently shown inverse relationships with the degree of skin pigmentation characteristics of the population. Another consistent finding is the positive association with exposure to solar ultraviolet radiation. However, the relative importance of solar exposure in the aetiology of BCC is probably more pronounced in other countries than in Finland, where the intensity of ultraviolet-B radiation is, for example, only one-ninth of the intensity encountered across the equator (Henriksen et al, 1989). Other risk factors of BCC include old age; male gender; ionizing radiation; chemical carcinogens such as polycyclic aromatic hydrocarbons, inorganic arsenic and psoralens; precursor lesions such as solar or actinic keratosis; and possibly also dietary factors and cigarette smoking (Scotto et al, 1996). These epidemiological risk factors may either be shared or not shared by family members.

This study suggests that about one-third of the overall variation in liability to BCC is explained by environmental factors common to the twins in a pair, with the remainder accounted for by individual environmental factors. The shared environmental factors could include very similar exposures to sun in the childhood, but also other factors that are shared by family members. The large proportion of twin pairs in which only one twin has BCC suggests that the relevant risk factors for BCC might be identified using a case–control approach in these discordant pairs.

### ACKNOWLEDGEMENTS

The Turku Finnish University Society, Academy of Finland and Finnish Cancer Foundation have supported this study.

### REFERENCES

Ahlbom A, Lichtenstein P, Malmström H, Feychting M, Hemminki K and Pedersen NL (1997) Cancer in twins: genetic and nongenetic familial risk factors. *J Natl Cancer Inst* **89**: 287–292

Carter DM (1989) Basal cell carcinoma. In *Dermatology in General Medicine*. Fitzpatrick TB, Eisen AZ and Wolff K (eds), pp. 759–765. McGraw-Hill: New York

Dahmane N, Lee J, Robins P, Heller P and Ruiz i Altaba A (1997) Activation of the transcription factor GLI 1 and the sonic hedgehog signalling pathway in skin tumours. *Nature* **389**: 876–881

Falconer DS (1981) *Introduction to Quantitative Genetics*. Longman Scientific & Technical: Harlow, UK

Finnish Cancer Registry (1997). *Cancer Incidence in Finland 1995, Cancer Statistics of the National Research and Development Centre for Welfare and Health*. Cancer Society of Finland publication no. 58: Helsinki

Hahn H, Wicking C, Zaphiropoulos PG, Gailani MR, Shenley S, Chidambaren A, Voreschovsky I, Holmberg E, Unden AB, Gillies S, Negus K, Smyth I, Pressman C, Leffell DJ, Gerrard B, Goldstein AM, Dean M, Toftgard R, Chenevix-Trench G, Wainwright B and Bale AE (1996). Mutations in the human homologue of Drosophila patched in the nevoid basal cell carcinoma syndrome. *Cell* **85**: 841–851

Heath AC, Neale MC, Hewitt JK, Eaves LJ and Fulker DW (1989). Testing structural equation models for twin data using LISREL. *Behav Genet* **19**: 9–35

Henriksen K, Stamnes K, Volden G and Falk ES (1989). Ultraviolet radiation at high latitudes and the risk of skin cancer. *Photodermatology* **6**: 110–117

Järvinen P, Kaprio J, Mäkitalo R, Koskenvuo M and Aho K (1992) Systemic lupus erythematosus and related systematic diseases in a nation-wide twin cohort. An increased prevalence of disease in mz twins and concordance of disease features. *J Intern Med* **231**: 67–72

Kaprio J, Sarna S, Koskenvuo M and Rantasalo I (1978) The Finnish Twin Registry: formation and compilation, questionnaire study, zygosity determination procedures, and research program. *Prog Clin Biol Res* **24B**: 179–184

Kaprio J, Koskenvuo M, Artimo M, Sarna S and Rantasalo I (1979) *The Finnish Twin Registry: Baseline Characteristics. Section I. Materials, Methods, Representativeness and Results for Variables Special to Twin Studies.* Kansanterveystieteen julkaisuja M47 (Publications of the Department of Public Health), Helsinki University Press: Helsinki

Karjalainen S, Salo H and Teppo L (1989) Basal cell and squamous cell carcinoma of the skin in Finland. Site distribution and patient survival. *Int J Dermatol* **28**: 445–450

Kramer KH, Lee MM and Scotto J (1977) Xeroderma pigmentosum: cutaneous, ocular and neurologic abnormalities in 830 published cases. *Arch Dermatol* **123**: 241–250

Lutzner MA (1978) Epidermolysplasia verruciformis: an autosomal recessive disease characterized by viral warts and skin cancer. A model for viral oncogenesis. *Bull Cancer* **65**: 169–182

Mackie RM (1986) Epidermal skin tumours. In *Textbook of Dermatology*. Rook A, Wilkinson DS and Ebling FJC (eds), pp. 1459–1504. Blackwell Science: Oxford

Mehta VR and Potdar R (1985) Bazex syndrome. Follicular atrophoderma and basal cell epitheliomas. *Int J Dermatol* **24**: 444–446

Neale MC (1997) *MX: Statistical Modelling*. Medical College of Virginia, Department of Human Genetics: Richmond, VA

Oro AE, Higgins KM, Hu Z, Bonifas JM, Epstein Jr EH and Scott MP (1997). Basal cell carcinomas in mice overexpressing sonic hedgehog. *Science* **276**: 817–821

Plomin R, DeFries JC, McClearn GE and Rutter M (1997) *Behavioral Genetics*. WH Freeman: New York

Sarna S, Kaprio J, Sistonen P and Koskenvuo M (1978) Diagnosis of twin zygosity by mailed questionnaires. *Hum Heredity* **28**: 241–254

Scotto J, Fears T, Kraemer KH and Fraumeni Jr JF (1996). Non-melanoma skin cancer. In *Cancer Epidemiology and Prevention*. Schottenfeld D and Fraumeni Jr JF (eds), pp. 1313–1330. Oxford University Press: Oxford

Southwick GJ and Schwarts RA (1979) The basal cell nevus syndrome: disasters occurring among a series of 36 patients. *Cancer* **44**: 2294–2305

Vitalino PD and Urbach F (1980) The relative importance of risk factors in non-melanoma carcinoma. *Arch Dermatol* **16**: 454–456

© Cancer Research Campaign 1998